# Factors Associated With Ambulation Status and Survival One Year After Conservative Management of Hip Fracture

**DOI:** 10.7759/cureus.64253

**Published:** 2024-07-10

**Authors:** Keisuke Nakamura, Tomohiro Sasaki, Marin Yokoyama, Takashi Kitagawa, Yuto Akashi, Masayuki Shimizu

**Affiliations:** 1 Department of Physical Therapy, Shinshu University, Matsumoto, JPN; 2 Department of Rehabilitation, Matsumoto City Hospital, Matsumoto, JPN; 3 Department of Orthopedics, Matsumoto City Hospital, Matusmoto, JPN

**Keywords:** mortality, older patients, independent walker, conservative treatment, hip fracture

## Abstract

Purpose: Few studies have investigated the factors associated with ambulation and survival over one year. Therefore, this study aimed to examine the factors that influence ambulation and survival rates in elderly patients who have undergone conservative management for hip fractures.

Materials and methods: This retrospective study included 74 ambulatory individuals aged 65 years or older prior to their injuries. One-year mortality and ambulatory status were assessed. Statistical comparisons of background and medical characteristics between groups of independent and non-independent walkers, as well as between survivors and mortalities, were performed using the Pearson chi-squared, Fisher exact, and Mann-Whitney U tests.

Results: The numbers of older patients able to walk independently, those not able to walk independently, and those with mortality at one-year post-injury after conservative management of hip fractures were 13 (18.3%), 35 (49.3%), and 23 (32.4%), respectively. Independent walkers one year after conservative treatment for hip fracture were younger (p=0.04) and less likely to have cognitive impairment (p=0.04) than non-independent walkers. The proportion of individuals with cognitive impairment was found to be lower among survivors than among mortalities (p=0.0098).

Conclusion: Cognitive decline may contribute to difficulties in walking independently and mortality at one year post-injury in this population.

## Introduction

In 2019, the global incidence of hip fractures had increased to 16.75 million, marking a significant surge of 166.4% since 1990, a trend closely associated with the aging of the worldwide population [1]. In particular, the number of patients aged ≥70 years with hip fractures increased, leading to increased mortality and worse functional prognosis [2,3]. Surgical intervention is the primary treatment option for patients with hip fractures. However, most patients with hip fractures are older and have multiple comorbidities that make surgical treatment challenging [2-4]. Consequently, conservative treatment is preferred in clinical practice.

Systematic reviews on the prognostic value of conservative therapy in older patients with hip fractures have reported high mortality rates and poor functional outcomes [5]; the pooled mortality rates at 30-day, six-month, and one-year intervals were 36%, 46%, and 60%, respectively. Only a small proportion of the surviving patients were able to mobilize after six months (9.6%) and one year (16.8%) [5]. Factors contributing to mobility at six months were younger age, fewer days to sit up, and higher cognitive function [6,7]. Only 5% of patients with advanced dementia following conservative therapy for hip fractures can ambulate six months post-injury [7]. However, few studies have investigated the factors associated with ambulation and survival at one year. In particular, few research has studied characteristics associated with walking independence one year following injury, with only 128 patients in three studies [6,8,9]. An investigation of the factors associated with long-term gait prognosis may provide useful information for the selection of appropriate treatment in older patients at high risk of surgical treatment following a hip fracture.

Therefore, this study aimed to investigate the factors associated with ambulation status and survival one-year post-injury after the conservative management of hip fractures. We hypothesized that age, cognitive function, and days to standing would be factors associated with ambulatory independence at one year, similar to a study conducted on patients after six months of hip fracture conservative therapy [6,7].

## Materials and methods

Study design and participants

This retrospective study was conducted at the Matsumoto City Hospital in Japan. Data were collected from April 2018 to April 2020, and follow-up data were collected until April 2021. This study included patients aged ≥65 years who received conservative treatment for femoral neck or trochanteric fracture. In addition, patients who were ambulatory before the injury were enrolled in the study.

This study was conducted in accordance with the guidelines outlined in the Declaration of Helsinki and approved by the Matsumoto City Hospital Ethics Committee on May 23, 2017 (17052304). Informed consent was obtained from all the participants. The study information, including the objectives, inclusion and exclusion criteria, and primary outcomes, was published in the publicly available University Hospital Information Network with the unique identifier UMIN000054199. This study was also conducted in accordance with the Strengthening the Reporting of Observational Studies in Epidemiology guidelines [10].

Measurement

Data on all-cause mortality and walking status one year after injury were obtained by telephone or written questionnaire; this included information on walking aids, walking distance, and amount of walking assistance. Walking status was classified as independent and non-independent walking. Independent walking was defined by gait Functional Independence Measure (FIM) ≥5, regardless of the walking aid used, and gait FIM <5 was defined as non-independent walking [11].

During hospitalization, the following data on patient background factors were collected: age, sex, body mass index, comorbidities (cardiovascular, respiratory, renal, and neurological diseases), Hasegawa Dementia Scale-Revised (HDS-R) [12] score assessed within one week of admission, pre-injury gait status (walking without assistive devices, walking with a one-point cane, using a walker, and using other assistive devices), and pre-injury residence. Data on medical factors, such as fracture type, complications (deep vein thrombosis, peroneal nerve palsy, and infection), time to first sitting up after surgery, and time to first standing after surgery, were recorded. In this study, the doctor's order was to start sitting exercises early in patients with femoral neck fractures and one week after the injury in patients with trochanteric fractures. In addition, we investigated the patients’ discharge destination, walking status, and length of hospital stay. Cognitive impairment was defined as an HDS-R score of ≥21 [12]. The sample size was not estimated as this was an exploratory study.

Statistical analysis

Continuous variables are presented as medians (interquartile ranges (IQRs)), whereas categorical variables are expressed as numbers and percentages. Factors, such as patient background and medical status, were compared between the independent and non-independent walking groups at one year after injury using the Pearson chi-squared test, Fisher exact test for count data, and Mann-Whitney U test. Additionally, the association between survival and mortality one year after injury was compared using the same statistical methods. The p-values were adjusted using Bonferroni correction to control for multiple comparisons [13].

All statistical analyses were performed with modified R Commander 4.3.2 (R Consortium, San Francisco, CA) (https://personal.hs.hirosaki-u.ac.jp/pteiki/research/stat/R/), which is a modified version of R Commander designed to add statistical functions frequently used in biostatistics. Statistical significance was set at p<0.05.

## Results

**Figure 1:**
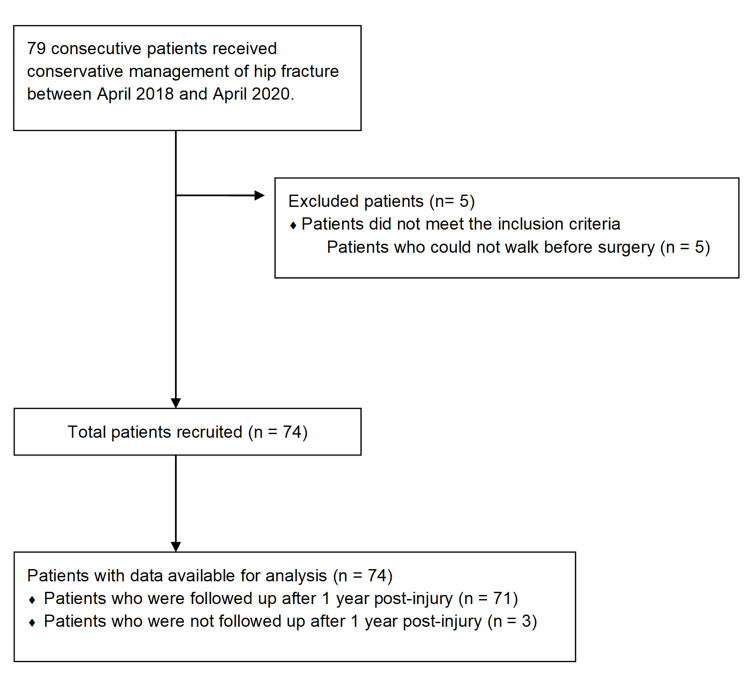
Patient recruitment and flow diagram

A total of 74 patients were included, but data were missing for three patients at one-year follow-up after injury (Figure 1). The median patient age was 91 (IQR, 6.8 years) (Table 1). Before the injury, 26 (35.4%) patients were able to walk without assistance; 10 (13.5%), walking with canes; and 38 (51.4%), walking with walkers. Among all the patients, 46 (67.1%) had cognitive impairment (Table 1). Additionally, half of the patients had cardiovascular disease as a comorbidity. The reasons for conservative treatment were medical factors, including cardiac disease and pneumonia, which were identified in 48 patients (64.9%). In 20 patients (27%), the decision for conservative treatment was based on patient and family preferences. However, the reason for this is unknown.

**Table 1:** Patient demographic data Data are presented as number (percentage) or median (IQR). NMV: Number of missing values, BMI: body mass index, GNRI: Geriatric Nutritional Risk Index, IQR: interquartile range.

Item	All, N = 74	NMV
Age (years)	91 (6.8)	0
Sex, n (%)		0
Male	22 (29.7)	
BMI (kg/m^2^)	19.8 (4.0)	2
Pre-fracture living situation, n (%)		0
Own home	56 (75.7)	
Nursing facility	18 (24.3)	
Pre-fracture walking status, n (%)		0
Walking without aids	26 (35.1)	
Walking with one point cane	10 (13.5)	
Walking with walker	38 (51.4)	
Medical comorbidities, n (%)		
Respiratory disease	9 (12.2)	0
Cardiovascular disease	37 (50.0)	0
Neurological disease	22 (29.7)	0
Fracture type, n (%)		2
Femoral neck	29 (39.2)	
Trochanteric	45 (60.8)	
Serum albumin level before surgery (g/dL)	3.4 (0.8)	4
GNRI	87.3 (13.8)	4
Cognitive impairment, n (%)	49 (67.1)	1
Complication during hospitalization, n (%)		
Peroneal nerve palsy	0 (0)	0
DVT	0 (0)	0
Infection	7 (9.5)	0
Time to first postoperative sitting (days)	6 (12)	7
Time to first postoperative standing (days)	15 (14)	6
Hospital stay (days)	59.5 (31.8)	4
Destination after discharge, n (%)		0
Own home	26 (35.1)	
Nursing home	41 (55.4)	
Death	7 (9.5)	
Mobility at one year postoperatively, n (%)		3
Independent walking at 1 year postoperatively, n (%)	13 (18.3%)	
Not independent walking at 1 year postoperatively, n (%)	35 (49.3%)	
Mortality at 1 year postoperatively, n (%)	23 (32,4%)	

**Figure 2:**
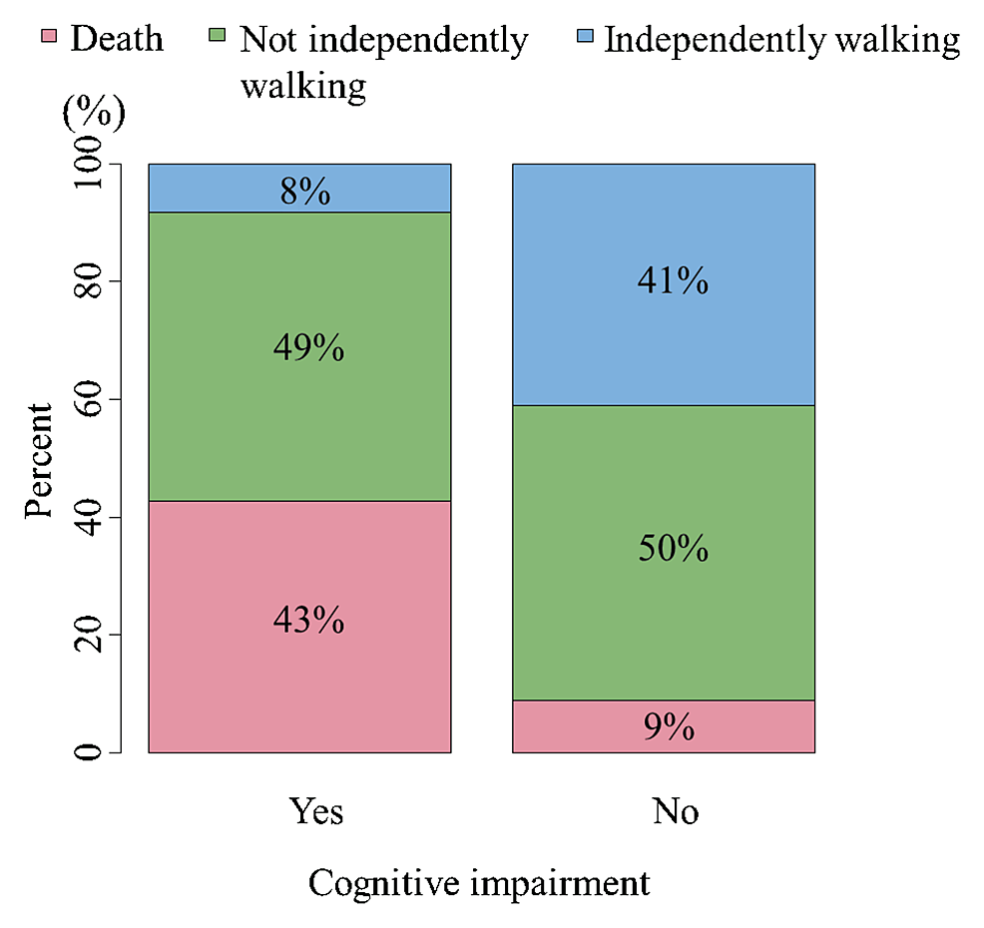
Cognitive impairment and walking status at one-year post-injury

**Figure 3:**
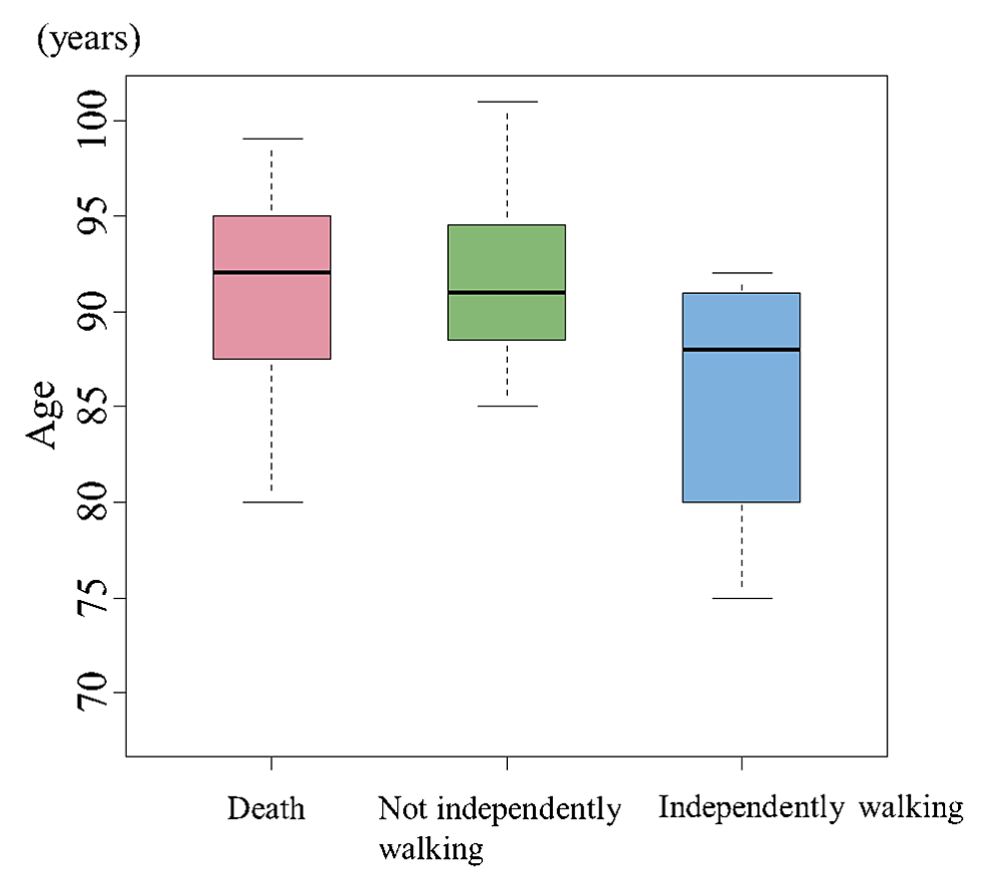
Age and walking status at one-year post-injury

The number (%) of patients walking and not walking independently at one-year post-injury were 13 (17.6%) and 35 (47.3%), respectively. Among all patients, 23 (31.1%) died within one-year post-injury. Patients walking independently one year after conservative treatment for hip fractures were younger (p=0.04), more likely to be men (p=0.04), and less likely to have cognitive impairment (p=0.04) than those who did not walk independently (Table 2). Patients who survived for one year after conservative treatment for hip fractures were more likely to have trochanteric fractures (p=0.0003) and no cognitive decline (p=0.0098) than those who died (Table 2). Patients with cognitive decline had a more unfavorable functional prognosis one year after conservative hip fracture therapy (Figure 2). Older age was associated with an increased risk of walking dependence and mortality (Figure 3). There was no significant difference in the number of days to first sitting up or standing after injury between patients who walked and did not walk independently one year after conservative hip fracture therapy.

**Table 2:** Comparison of patient background characteristics and rehabilitation factors between the groups Data are presented as number (percentage) or median (IQR). *P-values were adjusted by the Bonferroni method. NMV: number of missing values, GNRI: Geriatric Nutritional Risk Index, IQR: interquartile range.

Item	Independently walking, N = 13	Not independently walking, N = 35	Dead, N = 23	Independent vs Not independent walk, P-value*	Alive vs Death, P-value*
Age (years)	88 (11)	91 (6)	92 (7.5)	0.04	0.41
Sex: Male, n (%)	7 (53.8)	7 (20)	15 (34.8)	0.04	0.63
BMI (kg/m^2^)	20.3 (3.6)	20.1 (3.8)	17.8 (3.7)	0.89	0.05
Fracture type				0.13	0.0003
Femoral neck	1 (7.7)	10 (28.6)	16 (69.4)		
Trochanteric	12 (92.3)	25 (71.4)	7 (30.4)		
Medical comorbidities, n (%)					
Respiratory disease	1 (7.1)	4 (11.4)	4 (17.4)	0.71	0.41
Cardiovascular disease	5 (38.5)	17 (48.6)	15 (65.2)	0.53	0.13
Neurological disease	4 (30.8)	11 (31.4)	7 (30.4)	0.97	0.94
Serum albumin level before surgery (g/dL)	3.3 (0.8)	3.4 (0.5)	3.4 (0.9)	0.67	0.76
GNRI	88.5 (16.0)	88.6 (12.8)	84.0 (12.7)	0.91	0.14
Cognitive impairment, n (%)	4 (30.8)	24 (68.6)	21 (91.3)	0.04	0.0098
Time to first postoperative sitting (days)	6 (8.3)	8 (10.8)	5 (13)	0.99	0.33
Time to first postoperative standing (days)	7 (18)	16 (13)	13 (15)	0.28	0.56
Pre-fracture living situation, n (%)				0.85	0.92
Own home	10 (76.9)	26 (74.3)	17 (73.9)		
Nursing facility	3 (23.1)	9 (25.7)	6 (26.1)		
Pre-fracture walking status, n (%)				0.87	0.75
Walking without aids	5 (38.5)	11 (31.4)	9 (39.1)		
Walking with one point cane	2 (15.4)	5 (14.3)	2 (8.7)		
Walking with walker	6 (46.2)	19 (54.3)	12 (52.2)		

## Discussion

The results of this one-year follow-up of 74 patients who underwent conservative treatment for hip fractures indicate that, even in cases where conservative treatment was administered, walking ability can be restored in younger patients with good cognitive function. Several studies have investigated the prognosis of walking ability in patients undergoing conservative treatment for hip fractures [6-9,14-16]; 0%-48% of conservatively treated patients with hip fractures were ambulatory at six months or one-year post-injury [6,7,15,16]. However, a previous systematic review found little information on mobility at six months after injury (555 patients in two studies) and mobility at one year (128 patients in three studies) [5]. In this study, 74 patients who received conservative hip fracture therapy were followed up after one year, and 18.3% were able to walk independently, similar to the findings of previous studies [5]. Lim and Kwek indicated that younger age and shorter length of stay contributed to ambulation after six months [6]. Berry et al. showed that a lower severity of dementia was associated with a patient's ability to walk six months later [7]. In our study, one year after hip fracture, the patients who walked independently were younger than those who did not, and a greater proportion of those who walked independently did not show cognitive decline. Cognitive function may be a significant factor in the prognosis of gait after one year in patients with hip fractures undergoing conservative therapy, which is a novel finding; it may also be a significant factor in those who have undergone hip fracture surgery [17]. In contrast with the results of Lim and Kwek’s study [6], the number of days to first sit up after injury did not influence the ability to walk independently at one year in this study. The reason for this difference is that in this study, sitting and standing exercises were initiated according to the doctor's order.

In contrast, the one-year mortality rate in this study was 31%, which is lower than the mortality rates in previous studies (53.3-84.4%) [18-20]. In this study, the length of hospital stay was 59.5 days, which was considerably longer than that in previous studies [18-20]. Therefore, a longer inpatient management period may have contributed to the decrease in the mortality rate. Higher cognitive function and trochanteric fracture type were associated with a lower risk of mortality one year after injury. The association between cognitive decline and mortality in patients with hip fractures was consistent with the results of previous studies [21]. The higher mortality rate observed in this study for femoral neck fractures may be attributed to the inherent difficulty of bone fusion in such fractures, which may result in a reduced level of independence in mobility.

This study has several limitations that should be acknowledged. First, the small sample size may introduce confounding bias due to inadequate adjustment for confounders while examining the associations between influencing factors and functional prognosis. Another substantial limitation of this study is its reliance on data from a single institution. This dependency may introduce single-site bias given the institution’s unique characteristics. For instance, the specific demographics of the patient population, the level of medical staff expertise, and institutional treatment protocols may not mirror those prevalent in other healthcare settings. Consequently, these distinct attributes may limit the generalizability of the results to other institutions or diverse populations. Given the limitations of this study’s single-institution design, future research should be conducted across multiple institutions.

One of the major strengths of this study was its focus on functional outcomes one year after the conservative treatment of hip fractures, a topic that has been poorly addressed in the existing literature. This study fills a critical gap by providing valuable insights into long-term recovery trajectories following nonsurgical interventions for hip fractures. The results of this study are particularly important because they provide evidence-based guidance for clinicians in making decisions regarding the management of hip fractures, which may ultimately improve patient outcomes. The paucity of similar studies underscores the importance of this research in advancing our understanding of the efficacy of conservative treatments for hip fractures.

## Conclusions

The rate of walking independence after one year of conservative hip fracture therapy was 17.6%, and the rate of death was 31%. The results of this study are novel findings that suggest that cognitive decline may contribute to difficulties in walking independently after one year of conservative hip fracture therapy. Furthermore, age was a significant factor for gait reacquisition.
